# Compassion and Empathy in Basic Medical Science Teaching: A Suggested Model

**DOI:** 10.7759/cureus.20205

**Published:** 2021-12-06

**Authors:** Dana R Crawford

**Affiliations:** 1 Immunology and Microbial Disease, Albany Medical College, Albany, USA

**Keywords:** biomedical graduate students, medical students, teaching, empathy, compassion

## Abstract

Medical school education typically consists of two main student bodies: medical students and biomedical graduate students. For both groups, compassion and empathy represent a major component of future professional roles. For medical students, this takes the form of the all-important doctor-patient relationship and adherence to the Hippocratic Oath. For biomedical students, future research and teaching are often driven by the opportunity to contribute to treatments to help pain and suffering for those in need. For both groups, such positive contributions further extend to families, who often suffer emotional distress watching the health struggles of a loved one. Given the key role that compassion and empathy play here, including them as part of student educational development is important. Such focus, however, is limited - especially during the initial academic classroom years - with most time here dedicated to the learning of facts and foundational material. Given its importance in the future professional roles of these students, we posit that more can be done to introduce and reinforce the concept of compassion and empathy during the initial didactic course years. Modest but viable options exist for the introduction of these concepts as a part of basic teaching that will provide additional reinforcement of this all-important sensitivity for others. Here we present a model providing suggestions and recommendations for the integration of compassion and empathy in otherwise basic scientific teaching, and in a way that also includes progressive equality positions on social issues. While the focus here is medical school education since it represents this author’s expertise as well as a field where young trainees graduate to professional careers requiring compassion, it can potentially be applied to many other disciplines.

## Introduction

Compassion. Empathy. At their core, these concepts strongly overlap as they are both based on an attempt to understand how someone who is suffering feels and putting yourself in their shoes [1-3]. This commonality alone represents an admirable mindset for any individual and certainly for those in the medical profession, the focus of this paper. Technically, compassion takes empathy one step further by taking some sort of action to try to help the suffering. For those training for the biomedical profession, compassion and empathy represent a major component of future professional roles. This includes patient treatment and technological advances, which both aim to relieve pain and suffering for the patient as well as allay emotional distress in families watching the health struggles of a loved one. 

In the medical field, two major paths are medical school education toward an MD, and basic biomedical science graduate education toward a PhD. The typical medical school curriculum in the United States is four years long, with the first two years mainly classroom basic science education followed by two years in clinical settings. Biomedical science graduate education - dedicated to applying science education to healthcare - also includes classroom basic science education the first few years followed by a laboratory research emphasis. Given the key role that compassion and empathy play in the future careers of these individuals, including them as part of student educational development is important. Such focus, however, is limited, especially during the first few classroom (or self-directed) education years. We posit that more can be done to introduce and reinforce the concept of compassion and empathy during this time. 

Here we present a model providing suggestions for the integration of compassion and empathy into otherwise basic scientific teaching. Since the main emphasis during these years is the learning of basic facts and foundational material, such recommendations are modest so as not to interrupt the key learning focus. Nonetheless, even as subtle content, they represent new opportunities for integration of compassion and empathy, which reinforce the requirement of sensitivity for others within the medical field. Additionally, this new focus may also help raise awareness of social determinants of health.

## Materials and methods

In addition to this author’s many years of experience from teaching both medical and graduate students, discussions with medical and basic biomedical science graduate school faculty and students were carried out to assess exposure to compassion and empathy in otherwise basic scientific teaching during their initial academic classroom years. The aim of the model presented in this manuscript is to add material emphasizing these qualities without deflecting the central basic teaching presented, and to do so with a minimal amount of additional material necessary to convey these qualities. This additional information includes the selection of scientific role models based on their important contributions to the field and subject matter presented, and in a way that is inclusive of various demographics (here, gender and race) and social justice issues consistent with current societal emphasis.

Reassessment of the presentation of didactic material is also based on this author’s many years of teaching experience on a wide range of biomedical topics. As such, specific examples from my lectures that have long been presented and fine-tuned are included. These are not just as examples, but also as material I feel will likely benefit from the modifications presented in this manuscript while still getting the main basic science message across. This includes its likely continued utility as a part of newly evolving and future teaching techniques, such as small group and self-directed study topics. Organizations considered and ultimately recommended as part of this model (e.g., World Health Organization) were selected based on their humanitarian efforts, broad reach, longevity and successful outreach. Finally, this model is presented beyond just the patient to the broader context of families and communities where possible, since recognizing these groups as well is a further incentive for the success of this presented educational model. 

## Results

Medical students

Exposure to Compassion and Empathy During Early Classes 

For medical students, compassion and empathy are key components of their future doctor-patient relationship, and also their commitment to the Hippocratic Oath. However, integration of these concepts is somewhat limited during their first few pre-clinical years of study. Nonetheless, at least some are provided. Most notably, this includes a Health Care in Society longitudinal course that has dedicated classes to this topic, as well as addresses compassion and empathy in the context of other significant bioethical concerns in the medical field [4]. Additionally, there is a Clinical Skills longitudinal course in which students interact with actor patients in a clinical setting. In this course, a significant amount of the feedback regards how the students interact with the actor, with a specific focus on the compassion and empathy displayed by the student towards the actor patient. Other elective programs include the Healer’s Art [5,6], empathy skills training [7,8], narrative medicine [9,10], Balint groups [11,12], and compassion cultivation training courses such as through the Stanford Center for Compassion and Altruism Research and Education [13]. In addition and as cited in this paper, numerous articles have been written on compassion, empathy and feeling as part of the medical student learning experience. Clerkship and resident years provide additional opportunities through the focus on direct patient interactions.

Decrease in Compassion and Empathy During Medical School Education and Beyond 

Theoretically, the above training would be expected to improve compassion and empathy in students, and eventually translate to major benefit when these students turn professional. This is particularly important since compassionate and empathetic care has been shown to improve clinical outcomes for patients [2,3,8,14-18]. However, despite such training, a number of studies have reported a decrease in compassion and empathy in medical students as they progress through medical school due to various reasons [11,13,18-20]. Additionally, a lack or inconsistency of compassionate patient care by professional healthcare workers has been cited [15], in turn suggesting inadequate preparation during medical school.

Biomedical graduate students

For biomedical graduate students, compassion and empathy are related to their future research and teaching in that these individuals are often driven by the opportunity to contribute to treatments to help pain and suffering for those in need. Despite such goals, these trainees receive an almost shocking lack of exposure to the pain and suffering experienced by the very target (patient) population they are attempting to help! This is even far less than the modest amount of such experience provided to medical students as discussed above. As a prime example, Bioethics courses at least address patient experiences - and compassion and empathy for them - to some extent in the first few years of medical student education. Conversely, Bioethics education of graduate students shifts focus to research scientific integrity.

As cited throughout this paper, there are numerous publications discussing this issue for and about medical students. However, there's a stunning lack of such publications for biomedical graduate science learning. Certainly, many more of these publications would be expected for medical students given their direct interaction with patients and the eventual future physician-patient relationship. However, the lack of similar publications addressing graduate biomedical student compassion and empathy toward the very patient populations they are interested in helping seems to represent a lack of understanding of an important driving force behind biomedical graduate student study and interest. It also represents a lost opportunity in that such training, literature availability and patient understanding could contribute to even more successful patient treatment and diagnostic development. This is further exacerbated by a lack of patient interaction throughout basic graduate student study. 

Integration into scientific lectures

Approach

Based on the above background, we propose to introduce and/or improve the teaching of compassion and empathy in the formative first few years of medical school and biomedical science graduate education. As mentioned, this is to be done in a low-key way so as to not veer from the central teaching mission of conveying basic facts and information. This subtle inclusion in these typically “dry” basic lectures will reinforce the importance of compassion and empathy that might contribute further to the development of student mindset that will be so important in their future, and especially those affected by this (patients, families, and scientific discoveries focused on populations and families). Accordingly, we suggest the following additions and adjustments during this time as a working model that can be modified over time:

Strategic Use of Terminology

The use of specific lecture words that are associated with compassion and empathy is a simple and common-sense addition to subtly reinforce sensitivity for others. Suggested words and phrases here include:

- healing

- alleviate suffering

- understand how someone feels, and how that might feel for you

- sensitivity

- understanding

- care

- concern

- benevolence

For example, instead of “etoposide inhibits cancer cell division and thus is effective as an anti-tumor therapy”, this could simply be modified to “etoposide inhibits cancer cell division and thus is effective as an anti-tumor therapy, in turn helping alleviate suffering in patients and their families who have to witness this struggle”. Another option would be presenting information in a positive life-saving and life-affirming way; e.g., “p53 to the rescue” for understanding its central role and many processes, and how this knowledge can and is being exploited to save lives. Again, these are simple tweaks and are not expected to deflect the main basic science messages being taught. 

Use of Figures to Associate Major Findings With Present and Future Medical Benefit and Kindness

**Figure 1:**
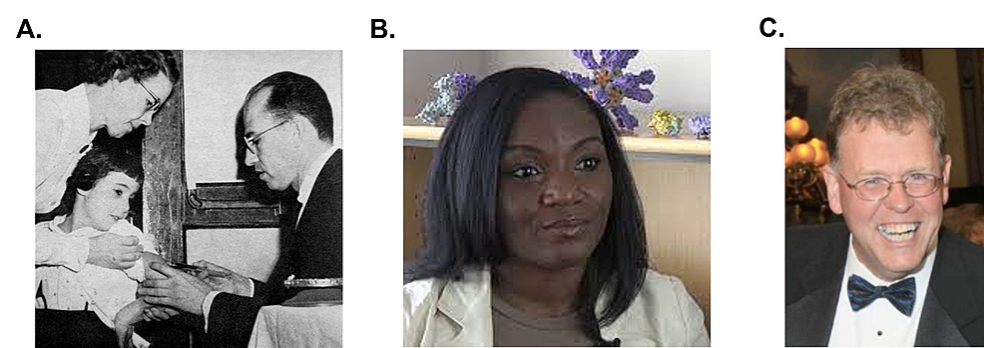
Important scientific contributors and humanitarians A. Dr. Jonas Salk and patient. B. Dr. Kizzmekia Corbett; per Creative Commons share [21]. C. Dr. Harm Velvis

Including presentation figures that connect individuals, patients, families and/or communities to key information helps reinforce compassion and empathy. Such figures can include key historical figures behind the medical discoveries or services, especially those whose important discoveries were driven by good-hearted humanitarian purposes and the people who benefit from this to emphasize the compassion and empathy connection. For example, as shown in Figure 1A, instead of simply teaching polio as an important breakthrough in our understanding of immunology and vaccine development, a picture of Dr. Jonas Salk and a child that he helped is added.

This immediately elicits a human connection, and a compassionate one at that. In addition, this can be presented along with information about the altruistic intent of these pioneering individuals serving as positive role models for compassion and empathy to further drive home these concepts, such as Dr. Salk’s decision to not patent or profit from this vaccine to maximize its global distribution (from the Salk Institute for Biological Studies website. History of Salk: About Jonas Salk). Dr. Kizzmekia Corbett (Figure 1B) is a more recent example of another researcher who has made important contributions to vaccine development, in this case, COVID-19 RNA and monoclonal antibody vaccines, and someone who has also done admirable community work to inspire youth in underserved communities [22,23]. Another example is Dr. Harm Velvis, founder of the Aim High Foundation to improve the lives of individuals and their families with Down syndrome (Figure 1C). Here, in addition to basic trisomy 21 and karyotype information being presented, Dr. Velvis can be shown as a connection to this benevolent cause. There are many other such examples. Revisiting historically important medical breakthroughs also elicits compassion and empathy in students by serving as a reminder of the extent of human suffering that existed before these discoveries.

Include Figures with Clinical Correlations

**Figure 2:**
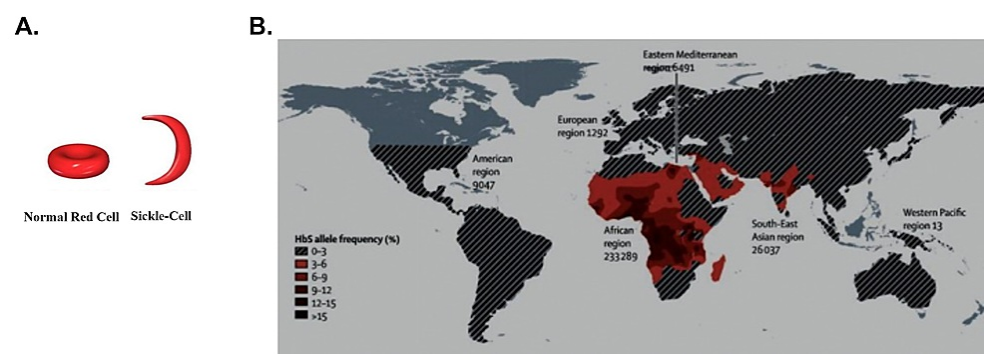
Sickle cell anemia A. Effect on red blood cell morphology; per Creative Commons share [21]. B. Geographical areas most affected; adapted and printed per Elsevier permission [24]

Clinical correlations are a tried-and-true teaching tool for connecting basic concepts to medical treatment. While they are often presented without figures, their whole point is to connect the medical scenario with patients and thus, a logical reason to show patients affected. While a simple step, this alone is likely to elicit a more compassionate and empathetic interpretation from students. Additionally, the manner the patients are presented can further this impact. For example, a family or even an entire community affected can be presented as part of this image. An example of this is given in Figure 2. Typically, I present sickle cell anemia as an extreme but nonetheless accurate example of a missense mutation as part of a DNA mutation lecture and also show an image of the altered red blood cell morphology that results (Figure 2A). This could be improved by adding Figure 2B to elicit greater compassion/empathy by simply showing the much greater risk of this devastating disease in certain parts of the world (equatorial Africa).

Include Organizations Known for Their Humanitarian Efforts in Figures

Organizations such as the World Health Organization, Gates Foundation and American Cancer Society are known for their humane health efforts so including them - even just logos - in figures immediately elicits a positive humanitarian connection. 

Extend Some Teachings to Include Ethical/Moral Concerns Such as Great Technological Advances

This overlaps somewhat with the Bioethics curricula, except here (for our basic science lecture recommendations), ethical concerns are only briefly mentioned as an extension of the main focus of the lecture - teaching students the complicated science behind the technology. Still, beneficial effects can be presented as well and as a reminder that such discoveries have led to treatment for such and such and reduction of pain and suffering. Beneficial … detrimental … in either case, it brings the human connection into play. Two great examples would be RNA sequencing (RNAseq) and CRISPR-Cas9 technology. For RNAseq, the main emphasis on the science behind this technology can be extended by the mention of the positive benefits of this technology (e.g., personalized/precision treatment depending upon RNA sequence) and then bioethical concerns (e.g., use of patient data to deny insurance or employment). The same holds for CRISPR benefits (a relatively fast, cheap and accurate gene therapy strategy) and bioethical concerns (e.g., human gene editing to alter appearance or intelligence). The concerning parts, even as brief mentions, also reaffirm concern for the well-being of others.

Add How Knowledge of Specific Pathways/Mechanisms Can Translate to Something Healing, and How This Can Further Reinforce Learning

Rather than just describing key pathways and mechanisms, which alone teach important didactic information, extend this to briefly include how this knowledge is exploited for healing. For example, instead of just “During cholesterol synthesis, HMG CoA-reductase is the key rate-controlling enzyme in the mevalonate pathway leading to cholesterol synthesis”, add “and inhibition of this key enzyme with statin drugs lowers serum cholesterol, in turn saving tens of thousands of American lives yearly”. Another example is the teaching of Direct Reversal DNA repair, which is somewhat confusing when describing photolyase as the first example identified for this type of repair, but an enzyme that is not present in humans. Here, adding that a sunscreen with added photolyase has been developed to reduce skin cancer again not only reinforces the healing perspective, but also provides a new way by which students can better remember that this historically important DNA repair enzyme is not synthesized by humans.

Dedicated Lectures From Those Suffering With Disease

While this inclusion would require greater time spent on a topic, it would rapidly connect all aspects of a given disease. This approach is actually used at our (Albany) medical college in the very first lecture of the first-year theme using cystic fibrosis as the disease. Here, when students learn about cystic fibrosis, having a patient with cystic fibrosis come in to describe their daily routine of chest physical therapy, pancreatic supplements, and high caloric intake reinforces the multiple body systems that the CFTR (cystic fibrosis transmembrane conductance regulator) gene mutation affects. Additionally, the inheritance pattern can be reviewed and brought to life if the patient is willing to share that aspect of their journey with cystic fibrosis. Again, this reinforces an important medical concept through a personal view - and the personal view from someone suffering from the disease elicits student compassion and empathy.

Social Justice Issues

A simple but effective use of our approach is to simultaneously remind or even raise student consciousness on important social issues, which is certainly important to medicine and patient treatment as well. An example is given above in Figure 1B for Dr. Kizzmekia Corbett, who has not only inspired youth in underserved communities but has also worked to gain the trust of minority populations hesitant about vaccinations [23]. Her simple addition, which already reminds students of important minority contributions to medicine AND introduces a name in a field where acclaim is lacking for African American contributors, can further advance social equality consciousness by - in this case - reminding students about the much higher rate of COVID-19 in minority populations. This in turn can also serve as a springboard for briefly referencing the social determinants of health. For example, the simple mention that African Americans have the highest death rate and shortest survival of any racial and ethnic group in the US for most cancers [25], and that this correlates with decreased access to health care and screening and other social inequities can be added just prior to returning to teaching the basic science behind carcinogenesis.

**Figure 3:**
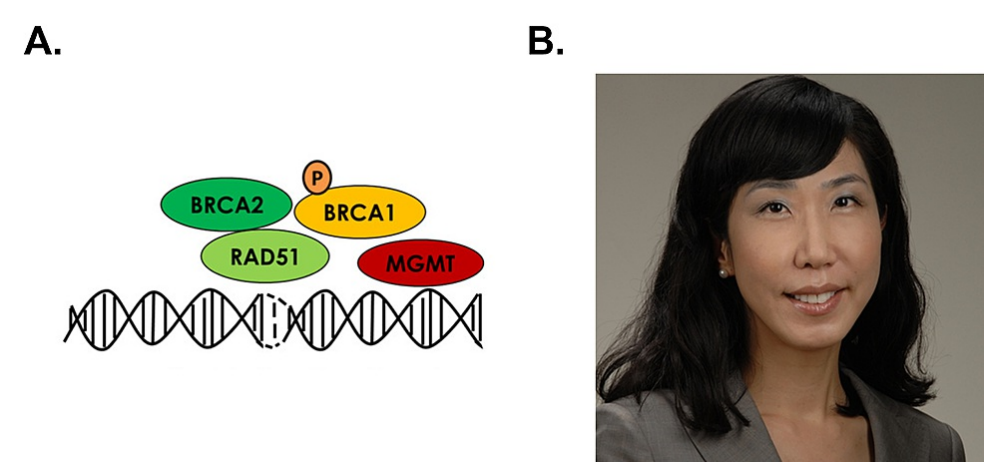
BRCA1/2 and Dr. Jung-Min Lee A. BRCA1, BRCA2 and RAD51 in double strand break repair; adapted and printed per Creative Commons share [27]. B. Dr. Jung-Min Lee; per Creative Commons share [28] BRCA: breast cancer gene

Another example is breast cancer gene (BRCA)1/2 mutations. This is much higher (almost 10-fold) in the Ashkenazi Jewish population (and in the black population as well to some extent) leading to a much greater risk of breast and ovarian cancers in these individuals [26]. Including this information during a presentation on the importance of BRCA1/2 genes/proteins in DNA repair and general recombination again casts a compassionate and sympathetic eye on a population with a long and sad history of anti-Semitic discrimination. As a further contribution to social justice awareness, it also underscores the importance of women-related research and patient treatment, which have historically been underrepresented due to sexism including underrepresentation in clinical trials. For example, instead of just stating “BRCA1 and BRCA2 are important accessory proteins in the action of Rad51 during double strand break repair” (as shown in Figure 3A), this could be extended to “BRCA1 and BRCA2 are important accessory proteins in the action of Rad51 during double strand break repair, and females with mutations in BRCA1/2 genes have greatly increased risk of breast and ovarian cancer; so high that many such carriers choose to have prophylactic surgery. This increased risk is especially prominent in the Ashkenazi Jewish population who are almost ten times more likely to carry these mutations”. Further social awareness here can be gained by adding the picture of a minority who has played an important role in researching and treating these cancers; in this case, Jung-Min Lee, MD (Figure 3B) of the National Institute of Cancer, who has carried out groundbreaking research in this field on BRCA1/2 mutation and PARP toward treating woman’s cancers [29,30]. 

Biomedical graduate student class

Since the lack of a patient connection is a glaring omission for biomedical graduate students as discussed above, a class or classes for this specific target audience could also be implemented that includes our strategy. Here, a common teaching approach for this group - a review of a recently published paper such as the biochemical mechanisms behind COVID-19 infectivity that constitutes the majority of the class - could be extended to include some specifics on the exact final vaccine being injected and follow up clinical reports on the actual number of lives saved by this technology. This will not only underscore how many lives are saved by research as a line of work, but also emphasize how the research these students do now and in the future - dedicated to the compassionate goal of healing - fits into the big picture medical puzzle that they will contribute to solving someday. It’s also a message that will help motivate the students even more. 

## Discussion

The above represents an impactful combination of simple tweaks to raise compassion, empathy, and social awareness toward affected patients/families/communities in students as a part of lecture material usually devoid of all this. Inclusion of individuals and their key contributions to science along with their involvement in social justice issues also connects the basic science with the faces of key personnel and the patients/families/communities they touch. This serves as a reminder that scientific breakthroughs in medicine are a team effort involving all races, ethnicities, and genders.

Including the above suggestions will also in turn logically stimulate at least some questions from students having to do with compassion/empathy simply because it’s part of the lecture content. Other related comments and discussions will follow in some cases. The transition to a greater emphasis on small groups and self-learning has the potential to accelerate this if students are exposed to more compassion and empathy from the above suggestions since their mindset will be more likely to include these concepts as they choose small groups and self-directed study topics. For medical students, it also provides a different manner of exposure that might help reverse the observed decrease in compassion and empathy that occurs as they progress through medical school.

While our approach is valuable for both medical and biomedical graduate students, the former at least have modest educational exposure dedicated to raising compassion and empathy in their field at listed above. Thus, our suggestions here are meant to integrate with these. Conversely, there is an inexcusable absence of such exposure for biomedical graduate students who are attracted to biomedical science not only because of their fascination with the field and its intellectual stimulation, but also by the hope of contributing to the prevention and treatment of individuals/families/communities who are suffering. A more proactive and compassionate/empathetic approach is needed to help these students better understand the patients their work and discoveries are intended to help as a part of graduate education.

## Conclusions

A new model is presented to introduce and reinforce the concept of compassion and empathy during the initial didactic course years. Modest but viable options exist for introduction of these concepts as a part of basic teaching that will provide additional reinforcement of this all-important sensitivity for others. It is also presented in a way that includes progressive equality positions on social issues. This represents a working model and as such, a foundation for additions, modifications and discussion. While many suggestions are provided, only a strategic number per lecture is recommended rather than comprehensively covering all so as not to detract from the main basic science information focus being presented. The overall intent is to raise awareness and provide additional reinforcement of these important concepts that represent such an important part of the future career of these students.
